# Squamous Cell Carcinoma of the Gallbladder With Recurrence at the Tumor Spillage Site Following Laparoscopic Cholecystectomy

**DOI:** 10.7759/cureus.101359

**Published:** 2026-01-12

**Authors:** Anupam Gupta, Kirk Hill, Harsha Polavarapu

**Affiliations:** 1 Surgery, Jackson Memorial Hospital, Miami, USA; 2 Pathology, Blessing Hospital, Quincy, USA; 3 Surgery, Blessing Hospital, Quincy, USA

**Keywords:** biliary tract malignancy, gallbladder cancer, incidental gallbladder carcinoma, laparoscopic cholecystectomy, peritoneal recurrence, squamous cell carcinoma, tumor spillage

## Abstract

Squamous cell carcinoma of the gallbladder is a rare and aggressive biliary malignancy, typically associated with locally invasive disease and poor outcomes, whereas most gallbladder cancers are adenocarcinomas staged primarily by depth of invasion. We report the case of a 76-year-old woman who presented with acute calculous cholecystitis and choledocholithiasis and underwent laparoscopic cholecystectomy followed by endoscopic retrograde cholangiopancreatography. During cholecystectomy for a severely inflamed and friable gallbladder, intraoperative gallbladder perforation with bile and gallstone spillage occurred. Final histopathology revealed a moderately differentiated squamous cell carcinoma of the gallbladder, pathological stage T1, confined to the lamina propria with negative surgical margins. Despite favorable initial staging, follow-up imaging at three months demonstrated local recurrence within the gallbladder fossa and peritoneal dissemination in the right paracolic gutter, consistent with early tumor recurrence. This case highlights the aggressive biological behavior of gallbladder squamous cell carcinoma and underscores the potential role of operative factors, including tumor spillage, in early recurrence despite apparently early-stage disease.

## Introduction

Gallbladder cancer is the most common malignancy of the biliary tract and is often difficult to detect at an early stage. Incidentally detected gallbladder cancer identified on histopathological examination following cholecystectomy has reported incidence rates ranging from 0.14% to 3%, varying by geographic region and patient-related risk factors such as gallstone disease [1]. Cholecystectomy performed for acute calculous cholecystitis is frequently associated with severe inflammation and friable tissue, making it challenging for the operating surgeon to distinguish benign inflammatory changes from an underlying malignancy intraoperatively [2].

Staging of gallbladder cancer, as outlined by most international guidelines, is primarily based on the depth of tumor invasion and is typically determined postoperatively on pathological examination [3]. While current guidelines recommend cholecystectomy alone as adequate treatment for early-stage (T1a) disease confined to the lamina propria, histologic subtypes such as squamous cell carcinoma are rare and may demonstrate distinct biological behavior. This case report aims to describe an unusual presentation of incidentally detected early-stage (T1a) squamous cell carcinoma of the gallbladder complicated by intraoperative gallbladder perforation and bile spillage during cholecystectomy for an inflamed gallbladder, followed by rapid local recurrence, and to highlight the potential implications of tumor biology and operative factors on recurrence risk despite favorable pathological staging.

## Case presentation

A 76-year-old woman with a body mass index (BMI) of 32.2 kg/m², a past medical history significant for gastroesophageal reflux disease managed with pantoprazole, and a non-smoking history presented to the emergency department with one day of acute-onset right upper quadrant abdominal pain. The pain was initially colicky, progressively became persistent and severe, and was associated with nausea and vomiting.

On clinical examination, the patient was tachycardic with a heart rate of 112 beats per minute, had localized right upper quadrant tenderness, and a positive Murphy’s sign. Initial laboratory evaluation revealed leukocytosis with a white blood cell count of 14 × 10³/µL (reference range 3.0-11.4) and abnormal liver function tests, including a normal total bilirubin and an elevated alkaline phosphatase of 303 U/L (reference range 44-147 U/L).

**Figure 1:**
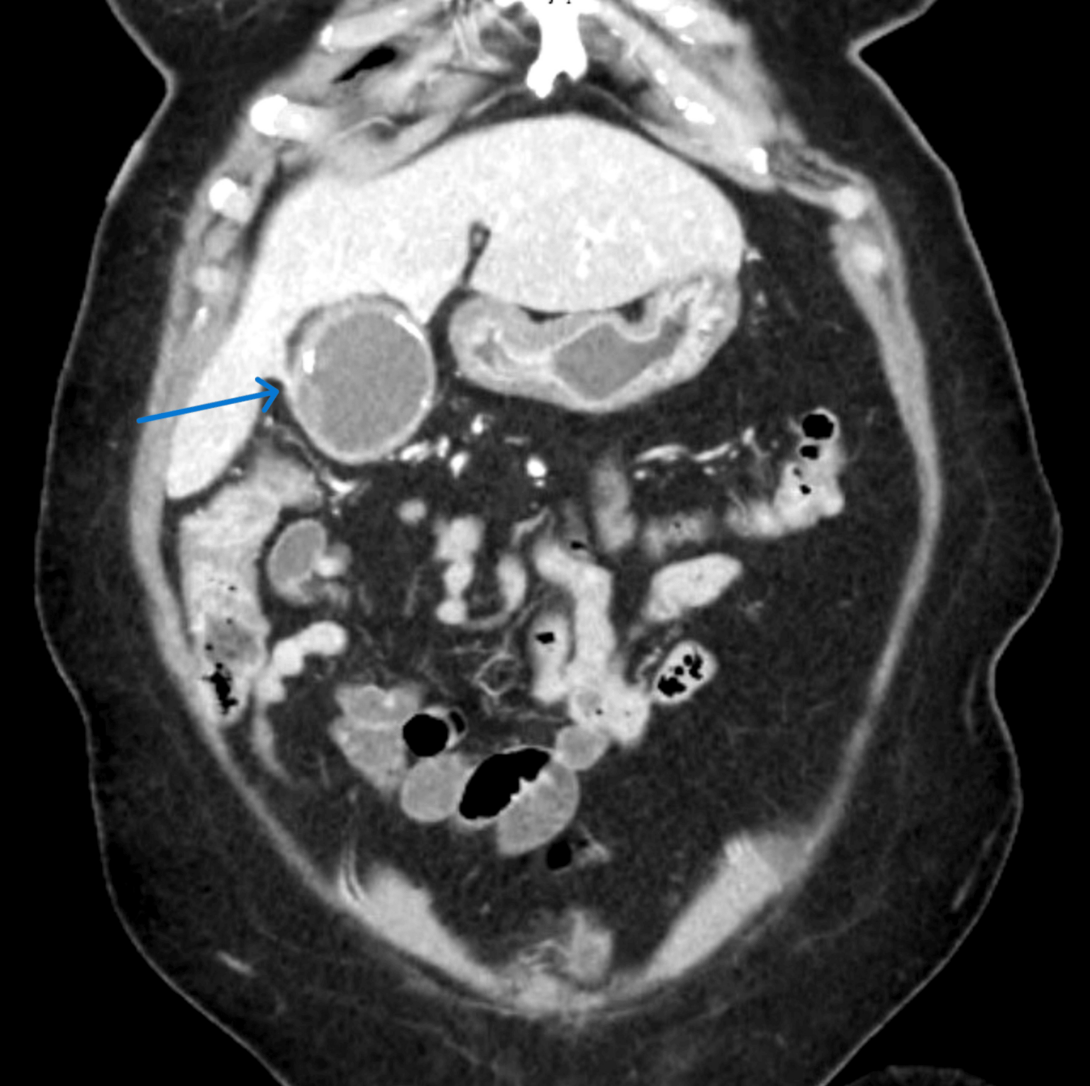
Preoperative computed tomography (CT) scan of the abdomen and pelvis demonstrating an inflamed gallbladder with gallbladder wall thickening and no evidence of metastatic disease.

Computed tomography (CT) of the abdomen demonstrated findings consistent with acute calculous cholecystitis (Figure 1), including gallbladder wall thickening, pericholecystic inflammatory changes, and dilation of the common bile duct to 1 cm. These findings were confirmed on ultrasound examination, which also demonstrated gallstones within the gallbladder.

The patient underwent a laparoscopic cholecystectomy with intraoperative cholangiography. Intraoperative cholangiography was performed in view of the dilated common bile duct noted on preoperative imaging. The procedure was carried out using a standard four-port technique. Intraoperative findings revealed an acutely inflamed, empyema of the gallbladder with a friable wall. During dissection, intraoperative gallbladder perforation occurred with spillage of bile, gallstones, and purulent material due to severe inflammatory changes. This was managed with immediate suctioning and copious irrigation using approximately 3 L of normal saline. The empyema and friable nature of the gallbladder were consistent with the severity of inflammation. Intraoperative cholangiography demonstrated filling defects within the common bile duct consistent with choledocholithiasis. The gallbladder was retrieved using an Endo-Catch bag, and a surgical drain was placed at the conclusion of the procedure. No other gross peritoneal or intra-abdominal lesions were identified intraoperatively.

The common bile duct stones were subsequently managed with endoscopic retrograde cholangiopancreatography, sphincterotomy, and stone extraction. The patient had an otherwise uneventful postoperative course, and the surgical drain was removed on postoperative day 3 due to minimal serous, non-bilious output.

**Figure 2:**
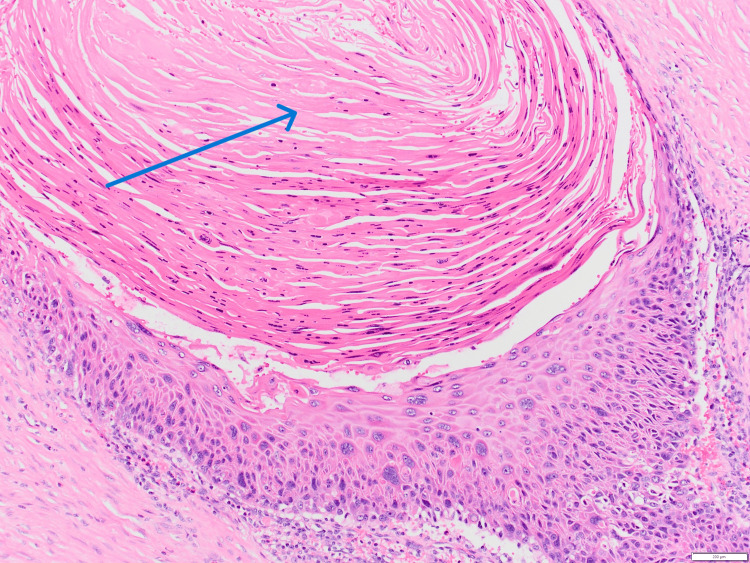
Hematoxylin and eosin-stained section demonstrating malignant squamous cells with prominent keratinization (arrow).

Histopathological examination of the gallbladder revealed a moderately differentiated squamous cell carcinoma (Figure 2) invading the lamina propria, involving the fundus and body of the gallbladder. The cystic duct margin was negative for malignancy. The tumor was classified as pathological stage pT1a (grade 2).

**Figure 3:**
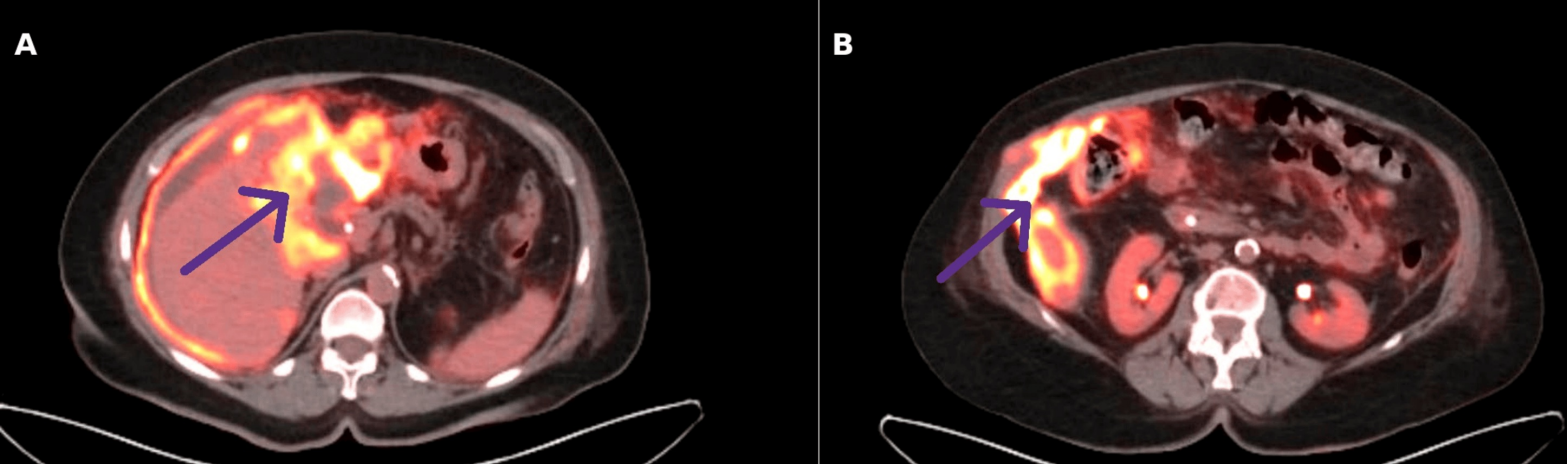
Positron emission tomography-computed tomography (PET-CT) demonstrating early tumor recurrence. (A) FDG-avid recurrence within the gallbladder bed at the index cholecystectomy site. (B) FDG-avid tumor deposit in the right paracolic gutter corresponding to the site of intraoperative spillage. FDG: fludeoxyglucose.

Given the incidental diagnosis of gallbladder carcinoma, the patient subsequently presented three months postoperatively with generalized weakness. In the setting of a known cancer diagnosis, positron emission tomography-computed tomography (PET-CT) was performed for further staging, which demonstrated local recurrence within the gallbladder fossa and peritoneal deposits in the right paracolic gutter, consistent with early tumor recurrence and peritoneal dissemination at sites corresponding to intraoperative spillage (Figure 3).

## Discussion

Gallbladder cancer is an uncommon malignancy but represents the most frequent cancer of the biliary tract. It occurs more commonly in older women than in men, with an approximate female-to-male ratio of 3:1 [4]. The disease originates from the epithelial lining of the gallbladder and may present either early or, more commonly, at an advanced stage. Adenocarcinoma accounts for approximately 90% of gallbladder cancers, with higher prevalence reported in regions such as India, Pakistan, South America, and other parts of the developing world [5,6].

Established risk factors include female sex, chronic gallstone-related inflammation, obesity, gallbladder polyps, *Salmonella *infection, toxin exposure (including aflatoxin and arsenic), and genetic predisposition [7]. Genetic alterations involving KRAS, TP53, and ERBB2 (HER2) have been associated with poorer prognosis [8].

Squamous cell carcinoma of the gallbladder is rare, accounting for approximately 1% of all gallbladder cancers, and is considered a more aggressive histological subtype. These tumors are typically identified at an advanced stage and most commonly arise in the fundus of the gallbladder. Histologically, squamous cell carcinoma is characterized by keratinization, sheets of malignant squamous cells, and the absence of glandular elements [9].

Gallbladder cancer is occasionally detected incidentally following a laparoscopic cholecystectomy performed for symptomatic cholelithiasis or acute cholecystitis. In such cases, final pathology is critical for diagnosis [1]. Staging and prognosis are primarily determined by the depth of tumor invasion and margin status. Increasing depth of invasion and positive margins necessitate further surgical intervention and consideration of adjuvant therapy [10].

The role of laparoscopic cholecystectomy in tumor dissemination has been debated. Intraoperative bile spillage does not alter the American Joint Committee on Cancer (AJCC) staging, which is based solely on the depth of invasion; however, it may increase the risk of peritoneal dissemination. Current evidence suggests that laparoscopic cholecystectomy does not confer a higher risk of tumor spread compared with open cholecystectomy, and routine port-site excision is not recommended [11,12].

Tumor markers such as CEA and CA 19-9 lack specificity but may be useful for surveillance and detection of recurrence [13].

Incidentally detected gallbladder cancer, as defined by the NCCN and International Hepato-Pancreato-Biliary Association (IHPBA), is managed based on tumor depth rather than histologic subtype or intraoperative spillage. For stage T1a disease confined to the lamina propria with negative margins and no metastatic disease, laparoscopic cholecystectomy alone is considered curative, with a reported five-year survival approaching 100% [13].

For tumors invading the muscularis propria or beyond, extended cholecystectomy with hepatic resection of segments IVb and V, regional lymphadenectomy, and, when indicated, bile duct resection is recommended. Advanced-stage disease is associated with significantly reduced survival, approximately 20% at five years [14].

Metastatic gallbladder cancer is generally managed with systemic chemotherapy, most commonly gemcitabine- or platinum-based regimens, although evidence supporting their efficacy in squamous cell carcinoma is limited. Squamous cell carcinoma of the gallbladder is associated with poorer outcomes compared with adenocarcinoma due to its aggressive biology and advanced presentation [15].

This case highlights an unusual and aggressive course, with early local and peritoneal recurrence following resection of incidentally detected early-stage squamous cell carcinoma.

## Conclusions

This case report describes early local recurrence and peritoneal dissemination following resection of an incidentally detected pT1a squamous cell carcinoma of the gallbladder, an exceptionally rare histological subtype. The observed pattern of rapid recurrence highlights a clinically notable presentation. The interplay of unusual tumor biology and intraoperative gallbladder perforation with bile and gallstone spillage may have contributed to this pattern, but causality cannot be established from a single case. These observations underscore the importance of meticulous surgical technique to minimize spillage when malignancy cannot be excluded and support the need for vigilant postoperative surveillance and further reporting of similar cases to better characterize outcomes in this rare entity.
